# Retrospective Analysis of Blood Inflammatory Biomarkers in Patients with Oral Lichen Planus: Neutrophil-to-Lymphocyte Ratio and Platelet-to-Lymphocyte Ratio

**DOI:** 10.3390/jcm13237490

**Published:** 2024-12-09

**Authors:** Pia López-Jornet, Francisco Parra-Perez, Priscila Pelaez, Eduardo Pons-Fuster

**Affiliations:** 1 Department of Dermatology, Stomatology, Radiology and Physical Medicine, Faculty of Medicine, University of Murcia, Hospital Morales Meseguer, Clinica Odontologica, Marques Velez S/N, 30008 Murcia, Spain; pacoppmurcia@gmail.com; 2 Biomedical Research Institute (IMIB-Arrixaca), University of Murcia, 30100 Murcia, Spain; pv.pelaezcruz@um.es; 3 Departamento de Anatomía Humana y Psicobiología, Faculty of Medicine and Odontology, Biomedical Research Institute (IMIB-Arrixaca), University of Murcia, 30100 Murcia, Spain; eduardo.p.f@um.es

**Keywords:** oral lichen planus, chronic inflammation, neutrophil-to-lymphocyte ratio (NLR), systemic immune-inflammation index, retrospective

## Abstract

**Objectives**: Oral lichen planus (OLP) is a potentially malignant disorder and a chronic inflammatory condition of an immune nature. The aim of this study was to investigate the association between immune-inflammatory biomarkers in patients with OLP and a control group. **Materials and Methods**: This was a retrospective study with 129 patients (62 with OLP and 67 controls) in which clinical and laboratory data were analyzed. The neutrophil-to-lymphocyte ratio (NLR), the platelet-to-lymphocyte ratio (PLR), the mean platelet volume (MPV) index, and the parameter of systemic immune-inflammation index (SII) were assessed. **Results:** In patients with OLP, the average time of progression was significantly longer when the condition manifested in the atrophic–erosive form (4.3 ± 3.2 years) as opposed to the reticular form (1.8 ± 0.9 years) (*p* = 0.018). With regard to NLR, no differences were found in terms of age (*p* = 0.346 (r = 0.08)), tobacco use (*p* = 0.807), sex (*p* = 0.088), alcohol consumption (*p* = 0.281), clinical form of OLP (*p* = 0.55), time of progression of OLP (*p* = 0.309 (r = −0.13)), and number of sites (*p* = 0.217). The same was observed for the systemic immune-inflammation index. **Conclusion:** The lack of significant statistical associations between the biomarkers and parameters (NLR, PLR, MPV, and SII index) in patients with oral lichen planus makes such parameters of very limited use in clinical OLP practice.

## 1. Introduction

OLP is a potentially malignant disorder and a chronic inflammatory condition of an immune nature that may occur in the skin and mucous membranes [1]. Its prevalence ranges between 0.1 and 4% and affects more commonly women between 30 and 70 years of age [2]. The etiology is unknown, but OLP pathogenesis suggests that T-lymphocyte-mediated autoimmune aggression is involved. Such T-lymphocytes target the epithelium and trigger apoptosis in basal keratinocytes, causing chronic inflammation [3,4,5]. Clinically, OLP appears as white, lacy papules and striae, leaving a variable erythema at the base [1,2]. There are different clinical types: reticular, erosive, atrophic, plaque, papular, bullous, etc.; currently, they are grouped into the following subtypes [3]: reticular OLP, characterized by Wickham’s striae, which are usually asymptomatic or slightly roughened, located on the posterior thirds of the buccal mucosa, and atrophic–erosive OLP [1,6], whose lesions tend to be painful and bleeding. OLP presents dynamic lesions that change in shape, appearance, and location. The most serious complication of oral lichen planus (OLP) is oral squamous cell carcinoma, with a malignant transformation rate of approximately 1.14%.

The risk of malignant transformations appears to be higher in OLP lesions with epithelial dysplasia compared to those without dysplasia (less than 1.5% transformation in non-dysplastic cases). The presence of epithelial dysplasia is the most significant factor in assessing the risk of malignancy in OLP. Some experts argue that cases of OLP that undergo malignant transformation might actually be lichenoid dysplasia rather than pure OLP. This highlights the need for standardized diagnostic criteria [1,2,3,4,5,6,7].

Therefore, continuous follow-up is recommended. However, the available evidence does not allow for specific recommendations regarding the frequency of said follow-up, though checking every 2–6 months is recommended for atrophic–erosive forms [6,7].

There exists growing interest in the search for biomarkers, and primarily serum inflammatory parameters, to predict disease aggressiveness, response to treatment, and prognosis [8,9]. The neutrophil-to-lymphocyte ratio (NLR) is a novel perspective marker of cellular immune activation, a valid index of stress and systemic inflammation, which opens a new dimension for clinical medicine to better understand the biology of inflammation, as well as the coupling and antagonism between innate and adaptive immunity and its clinical consequences [8].

Today, NLR has shown prognostic value in almost all medical disciplines as a reliable and easily accessible marker of the immune response to several stimuli in cardiovascular and inflammatory diseases, infections, and various types of cancer [8,9,10,11]. The neutrophil-to-lymphocyte ratio is considered a systemic inflammatory marker that correlates with disease severity [8]. There are currently few studies investigating the association between OLP and indices based on the systemic inflammatory response, and these works even show conflicting results [12,13,14,15,16,17,18,19]. The aim of this study was to determine the values for NLR, platelet-to-lymphocyte ratio (PLR), mean platelet volume (MPV) index, and systemic immune-inflammation (SII) index observed in an adult population of patients with OLP and a control group.

## 2. Materials and Methods

This study was conducted in accordance with the Declaration of Helsinki and approved by the Bioethics Committee for Research of the University of Murcia, Spain (ID: 4210/2022). This retrospective observational study follows the STROBE (Strengthening the Reporting of Observational Studies in Epidemiology) recommendation guidelines. Our study included patients who were seen at the Oral Medicine dental clinic at the University of Murcia.

The inclusion criteria were men and women >18 years of age with the presence of lichen planus in their oral mucosa (OLP) who met the clinical and histopathological criteria in accordance with the criteria by Van der Waal and Van der Meij [20].

The exclusion criteria were the following: minors, pregnant women, presence of epithelial dysplasia, oral lichenoid lesions related to medication and dental materials, graft-versus-host disease, radiotherapy or oncological chemotherapy, coagulopathies, infection findings (such as a white blood cell count > 12,000/mL or neutrophils > 70%), hematological diseases (hemoglobin level < 12 g/dL or >18 g/dL), and autoimmune diseases (such as Behçet’s Disease or Hashimoto’s Thyroiditis). In addition, those patients without a biopsy or with no concordance between clinical feature and pathology were excluded. The control group consisted of patients who were examined in the same Department of Oral Medicine at the Dental School, with the same age, sex characteristics, and other diseases or conditions (oral fibromas, racial pigmentations, salivary cysts). A total of 129 patients (62 with OLP and 67 belonging to the control group) were included.

**Figure 1:**
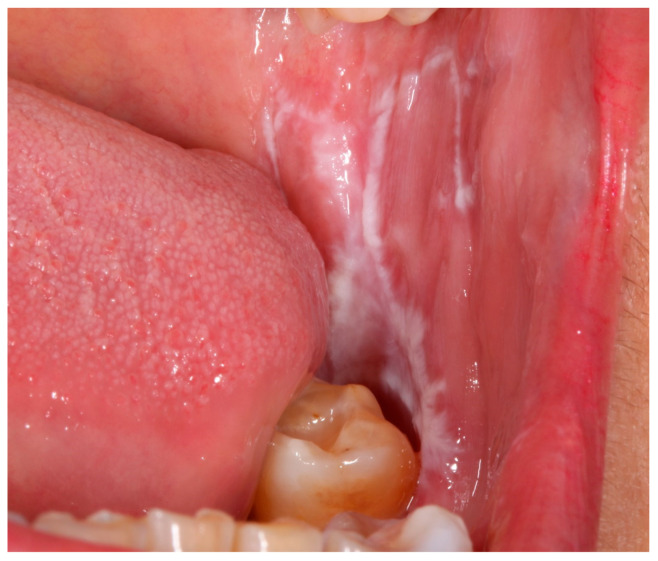
Oral lichen planus reticular.

**Figure 2:**
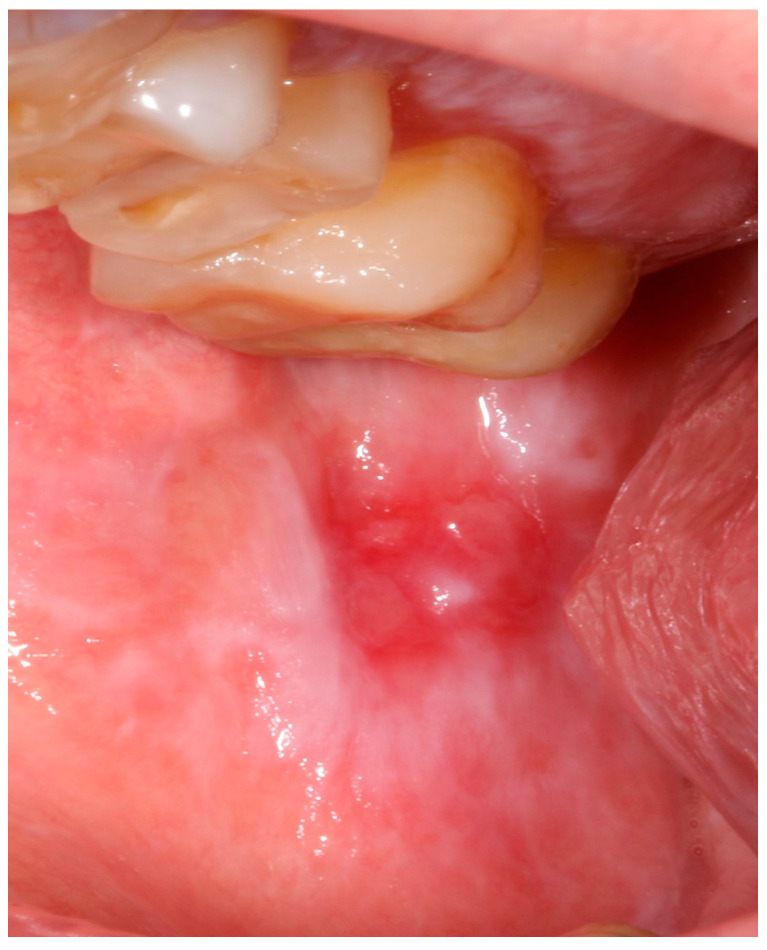
Oral lichen planus atrophic–erosive.

The protocol for the clinical findings included the following: sex, age, tobacco use (yes/no), former smoker, alcohol consumption (yes/no), and former alcohol drinker. The characteristics of OLP included clinical presentation, and the following information was additionally recorded: the presence of extraoral features (on skin, genitals, and scalp), the predominant type of OLP (reticular or atrophic–erosive) (Figure 1 and Figure 2), the number of intraoral sites, and the time of progression.

Blood samples were obtained by venipuncture, and complete blood cell indices and inflammatory markers were calculated. Blood, neutrophil, lymphocyte, and platelet counts were used in the present study, along with the reference ranges from our definition of systemic inflammatory markers.

Each systemic inflammatory score was calculated as follows: NLR = absolute neutrophil count (×10^9^/L)/absolute lymphocyte count (×10^9^/L); PLR = absolute platelet count (×10^9^/L)/absolute lymphocyte count (×10^9^/L); and SII (systemic immune-inflammation index) = absolute neutrophil count (×10^9^/L) × absolute platelet count (×10^9^/L)/absolute lymphocyte count (×10^9^/L). Meanwhile, the mean platelet volume (MPV) represented the average platelet size, reflected platelet stimulation and production rates, and indicated platelet function and the activation of inflammation.

## 3. Statistical Analysis

Data were analyzed by means of the SPSS version 12.0 statistical package (SPSS^®^ Inc., Chicago, IL, USA), and a descriptive study of each variable was performed. Inferential analysis includes the following tests: Chi^2^ test, which measures the degree of association between two categorical variables; a two-sample *t*-test, utilized to determine whether or not two population means are equal, thus confirming whether they could be assumed to come from the same distribution; and an ANOVA test, which compares variances across the means of two or more independent samples in order to detect significant differences between the group means. This type of analysis is generally applied when there is a continuous variable and a categorical variable with more than two categories, while Pearson’s R correlation is used to evaluate the linear association between two continuous variables. In this context, statistical significance was accepted for *p* ≤ 0.05.

## 4. Results

A total of 129 patients were included in this study: 105 women (81.4%) and 17 men (18.6%), with an average age of 59.3 ± 12.7 years and an age range of 17 to 86 years. Table 1 shows that no significant differences between the two groups with regard to demographic characteristics (age, sex, smoking habits, and alcohol consumption) and lichen planus characteristics were found.

In patients with OLP, disease progression was, on average, 2.4 ± 1.9 years, with a range of 1 to 10 years. In fact, the average time of progression was found to be significantly longer when the condition manifested in an atrophic–erosive form (4.3 ± 3.2 years) as opposed to a reticular form (1.8 ± 0.9 years) (*p* = 0.018). On the other hand, an extraoral site was found in 12.9% of patients.

Table 2 shows the laboratory variables under study. No significant differences between the two study groups were observed, but there was dissimilarity in the leukocyte levels. For the test group, the mean leukocyte levels were 6.14, increasing up to 6.64 (*p* = 0.054) when the control group was assessed.

Regarding NLR, no differences were found in terms of age (*p* = 0.346 (r = 0.08)), tobacco use (*p* = 0.807), sex (*p* = 0.088), alcohol consumption (*p* = 0.281), clinical form of OLP (*p* = 0.55), time of progression of OLP (*p* = 0.309 (r = −0.13)), and number of sites (*p* = 0.217).

In analyzing PLR, differences concerning tobacco use were observed (*p* =0.048). However, no differences regarding age (0.977 (r = 0.00)), sex (*p* = 0.088), alcohol consumption (*p* = 0.537), clinical form of OLP (*p* = 0.731), time of progression of OLP (0.206 (r = −0.17)), and number of sites (*p* = 0.760) were found. With regard to the systemic immune-inflammation (SII) index (10^9^/L), there were no differences in terms of age (*p* = 0.934 (r = 0.01)), tobacco use (*p* = 0.955), sex (*p* = 0.067), alcohol consumption (*p* = 0.059), clinical form of OLP (*p* = 0.609), time of progression of OLP (0.124 (r = −0.19)), and number of sites (*p* = 0.148 (Table 3)).

## 5. Discussion

Oral potentially malignant disorders (OPMDs), including oral lichen planus (OLP), are associated with the risk of transformation into oral squamous cell carcinoma (OSCC) [2,7,8]. Inflammation plays a key part in tumour development, since it is considered the seventh hallmark of carcinogenesis [1,6,21]. In this study, no significant differences between the immuno-inflammatory markers in patients with OLP and the control group were observed; these results are in line with other authors [11,12,16]. Ozlu et al. [19] found no statistically significant difference between participants with lichen planus (LP) and healthy participants; it is important to highlight that their study focused on cutaneous lichen planus (dermatological) rather than oral lichen planus. Zare et al. [12] did not investigate the NLR value, but they evaluated the neutrophil and lymphocyte counts separately and found that there was no significant difference between these hematological parameters.

NLR is a simple, rapid-response, and easy-to-obtain parameter of stress and inflammation. It is also a novel perspective marker of cellular immune activation, an index of stress and systemic inflammation, which opens new possibilities for clinical medicine [8,9]. NLR unites in a single value the occurrence of increased numbers of circulating neutrophils (involved in a much more rapid response) and decreased lymphocyte counts (long-term response of the immune system), making it very useful as a diagnostic tool [10,11], since these measures also correlate with disease severity. Hatice Ataş et al. [17] showed that patients with LP exhibit higher N/L ratios than controls, indicating that this could be associated with a systemic inflammatory process in LP. Thus, the N/L ratio may be recommended for the estimation of disease severity and treatment options. Ertem AG et al. [18] also found that this value was higher in patients who were sick compared to controls and Tosun V et al. [22] observed significant differences between patients with LP and controls in terms of NLR and PLR, as well as of the duration of LP.

The results presented in this study are in line with An et al. [16], who calculated NLR, PLR, and MPV values in patients with LP and concluded that NLR and PLR were not appropriate parameters to show inflammation in LP.

Although OLP etiology and pathogenesis are not fully understood, there exists abundant evidence that the T cell-mediated immune response plays a key role in the onset and development of this disease [4,5].

The role of p63 in the pathogenesis of oral lichen planus (OLP) has been the subject of active speculation, especially since Ebrahimi M et al. detected circulating antibodies against this protein in the serum of patients with OLP. Moreover, p63 deficiency along with an increase in p53 compromise cellular repair, increasing the risk of malignant transformation in severe or persistent cases. Correcting p63 deficiency could reduce this risk and improve the effectiveness of personalized treatments in patients with OLP [23,24].

Various molecular markers, including p63, HSP90, Ki-67, COX-2, TNF-α, and matrix metalloproteinases (MMPs), have shown relevance in oral lichen planus (OLP) by providing insights into its development and potential malignant progression. For instance, HSP90, often overexpressed in OLP, may be linked to chronic inflammatory stress and neoplastic transformation, while Ki-67 is associated with increased epithelial cell proliferation. COX-2, TNF-α, and MMPs are implicated in inflammatory, angiogenic, and tissue degradation processes. Techniques such as ELISA and RT-PCR can facilitate their detection in blood. Future research should focus on validating their diagnostic utility and developing non-invasive strategies for managing this condition [3,4,6,21,25,26,27,28,29].

The concept of NLR has generated new and profound insights into the dynamic course of the immuno-inflammatory response as a reaction between innate and adaptive cellular immune systems throughout various pathological processes [10,21,22,23,25,26,27,28,29]. Yamamoto et al. [24] reported that both lymphocyte and neutrophil functions were impaired and that cellular immunosuppression was a pathological feature of oral lichen planus (OLP). In this study, no significant differences among the different clinical forms of OLP were found regarding indices based on systemic inflammatory response, i.e., neutrophil-to-lymphocyte ratio (NLR), platelet-to-lymphocyte ratio (PLR), and mean platelet volume (MPV). Accordingly, the lack of significant association to the oral mucosa may restrict clinical use.

MPV provides information about the role and activity of platelets, indicates inflammatory processes, and is capable of monitoring activity in numerous diseases, e.g., patients with recurrent aphthous stomatitis, Behçet’s Disease, and psoriasis [30,31]. The study conducted by An et al. [16] found that the MPV levels in patients with LP were significantly higher than those in healthy controls, but without a significant relationship between MPV, oral mucosa, and nails. Yao et al. [14] observed increased MPV levels in Chinese patients with OLP despite no significant differences in platelet counts, whereas Ozlu et al. [19] showed that patients with dermal LP exhibited decreased MPV levels compared to healthy controls.

The systemic immune-inflammation (SII) index, which combines the three aforementioned cell lines, obtains good results [10] as it theoretically shows immune-inflammatory imbalance more accurately. In our case, it was not possible to observe significant differences in the clinical form of OLP (*p* = 0.609), time of progression of OLP (0.124 (r = −0.19)), and number of sites (*p* = 0.148). This could have been due to the fact that, in oral sites, the magnitude of the inflammatory response is lower, thus exhibiting a milder clinical presentation.

The possible explanation for the lack of significance may lie in the localized nature of oral lichen planus (OLP), which is confined exclusively to the oral cavity. Unlike other autoimmune diseases characterized by systemic manifestations or multi-organ involvement, OLP typically presents as a condition limited to the oral mucosa [6,12]. This specific localization may lead to immune responses that are more subtle or highly compartmentalized, not reflected in systemic parameters. This hypothesis aligns with previous studies suggesting that OLP is driven by the localized activation of T cells and cytokines at the affected site, with minimal or no systemic impact.

Major limitations in the present study were its retrospective, single-centre design and relatively low number of participants.

However, these limitations do not diminish the importance of this study, as it provides a foundational basis for future, larger-scale, multi-centre research. One of the key contributions of this study is the application of hematological indices as potential immuno-inflammatory biomarkers in patients with OLP, representing an innovative approach which opens new avenues for research in this area. Additionally, it is noteworthy that all the patients included in this study were accurately diagnosed through a biopsy of their oral lesions, ensuring the reliability of the collected data. It is worth mentioning that existing studies on hematological biomarkers in oral lichen planus are limited, and some previous reports show discrepancies in their findings [9,13,16,17]. These inconsistencies are likely due to differences in methodologies, participant selection criteria, and variations in laboratory protocols. This study not only provides new evidence in this field but also emphasizes the need for standardized methodologies in future research to facilitate the comparability of the results.

## 6. Conclusions

This work represents an important step toward the use of hematological indices as tools for better understanding OLP. Future research with larger sample sizes, prospective designs, and multi-centre approaches will help confirm and expand upon these findings, contributing to the development of more effective diagnostic and therapeutic strategies.

## Figures and Tables

**Table 1:** Homogeneity of the study groups in terms of demographic characteristics and toxic habits (Student’s *t*-test and Pearson’s χ^2^).

Characteristics	Control Group	Lichen Planus	*p*-Value
	(*n* = 67)	(*n* = 62)	
Age: mean ± SD **	60.03 ± 13.7	58.1 ± 11.5	0.33
Sex: *n* (%)			
Male	11 (16.4%)	13 (21%)	0.662
Female	56 (83.6%)	49 (79%)	
Smoking behaviour: *n* (%)			
No	37 (56%)	39 (62.9%)	0.645
Yes	7 (10.6%)	7 (11.3%)	
Ex-smoker	22 (33.3%)	16 (25.8%)	
Alcohol consumption: *n* (%)			
No	50 (76.9%)	44 (71.9%)	0.721
Yes	2 (3.1%)	3 (4.8%)	
Former alcohol drinker	13 (20%)	15 (24.2%)	
Characteristics of OLP			
Reticular		49 (79%)	
Atrophic–erosive		13 (21%)	
Location number			
2		47 (76.1)	
≥3		14 (23.8%)	
Extraoral features			
No		54 (87.1%)	
		8 (12.9%)	
Evolution (years)		2.4 ± 1.9	

OLP = oral lichen planus; and ** SD = standard deviation.

**Table 2:** Laboratory results of the patients (control and oral lichen planus).

Parameter	Control (*n* = 67)	Oral Lichen Planus (*n* = 62)	*p*-Value
Leucocytes (10^9^/L), mean (SD)	6.64 ± 1.48	6.14 ± 1.45	0.054
Neutrophiles (10^9^/L), mean (SD)	3.40 ± 0.92	3.15 ± 0.9	0.124
Lymphocytes (10^9^/L), mean (SD)	2.41 ± 0.78	2.19 ± 0.63	0.083
Platelet (10^9^/L), mean (SD)	247 ± 63	234.9 ± 52.6	0.211
MPV	9.54 ± 1.63	9.55 ± 1.37	0.069
NRL	1.50 ± 0.5	1.54 ± 0.63	0.725
PLR	108.7 ± 32.5	113.7 ± 38	0.438
SII (10^9^/L), mean (SD)	363 ± 142.3	358 ± 161.7	0.855

MPV = mean platelet volume; NLR = neutrophil-to-lymphocyte ratio; PLR = platelet-to-lymphocyte ratio; SII = systemic immune-inflammation index (10^9^/L); and SD = standard deviation.

**Table 3:** Results of the patients with reticular and atrophic–erosive lichen planus.

Parameter	Lichen Planus, Reticular (*n* = 49)	Lichen Planus, Atrophic–Erosive (*n* = 13)	*p*-Value
Evolution, years	1.8 ± 0.9	4.3 ± 3.2	0.018
Leucocytes (10^9^/L), mean (SD)	6.14 ± 1.7	6.14 ± 1.43	0.997
Neutrophiles (10^9^/L), mean (SD)	3.14 ± 0.91	3.19 ± 0.9	0.880
Lymphocytes (10^9^/L), mean (SD)	2.19 ± 0.58	2.22 ± 0.81	0.850
Platelet (10^9^/L), mean (SD)	235 ± 48.9	234.5 ± 67.0	0.91
MPV, mean (SD)	9.68 ± 1.42	9.16 ± 1.16	0.96
NRL, mean (SD)	1.50 ± 0.5	1.66 ± 091	0.55
PLR, mean (SD)	112.5 ± 34.3	117.7 ± 49.9	0.73
SII (10^9^/L), mean (SD)	350.7 ± 140.4	385.7 ± 230.2	0.339

NLR = neutrophil-to-lymphocyte ratio; PLR = platelet-to-lymphocyte ratio; MPV = mean platelet volume; SII = systemic immune-inflammation index; and SD = standard deviation.

## Data Availability

The data presented in this study are available upon request from the corresponding author. To protect privacy, the data are not publicly available.
